# The SARS-CoV-2 B.1.1.529 Omicron virus causes attenuated infection and disease in mice and hamsters

**DOI:** 10.21203/rs.3.rs-1211792/v1

**Published:** 2021-12-29

**Authors:** Michael Diamond, Peter Halfmann, Tadashi Maemura, Kiyoko Iwatsuki-Horimoto, Shun Iida, Maki Kiso, Suzanne Scheaffer, Tamarand Darling, Astha Joshi, Samantha Loeber, Stephanie Foster, Baoling Ying, Bradley Whitener, Katharine Floyd, Michiko Ujie, Noriko Nakajima, Mutsumi Ito, Ryan Wright, Ryuta Uraki, Rong Li, Yuko Sakai, Yanan Liu, Deanna Larson, Jorge Osorio, Juan Hernandez-Ortiz, Karl Čiuoderis, Kelsey Florek, Mit Patel, Allen Bateman, Abby Odle, Lok-Yin Wong, Zhongde Wang, Venkata Viswanadh Edara, Zhenlu Chong, Larissa Thackray, Hiroshi Ueki, Seiya Yamayoshi, Masaki Imai, Stanley Perlman, Richard Webby, Robert Seder, Mehul Suthar, Adolfo Garcia-Sastre, Michael Schotsaert, Tadaki Suzuki, Adrianus Boon, Yoshihiro Kawaoka, Daniel Douek, Juan Moliva, Nancy Sullivan, Matthew Gagne, Amy Ransier, James Case, Trushar Jeevan, John Franks, Thomas Fabrizio, Jennifer DeBeauchamp, Lisa Kercher, Patrick Seiler, Gagandeep Singh, Prajakta Warang, Ana S. Gonzalez-Reiche, Emilia Sordillo, Harm van Bakel, Viviana Simon

**Affiliations:** Washington University in Saint Louis; Influenza Research Institute, Department of Pathobiological Sciences, School of Veterinary Medicine, University of Wisconsin, Madison, WI, USA; University of Wisconsin; University of Tokyo; Department of Pathology, National Institute of Infectious Diseases, Tokyo; Institute of Medical Sciences, University of Tokyo; Washington University in St. Louis; Washington University School of Medicine; Washington University School of Medicine; University of Wisconsin-Madison; Center for Childhood Infections and Vaccines of Children’s Healthcare of Atlanta, Department of Pediatrics, Emory Vaccine Center, Emory University School of Medicine; Washington University School of Medicine; Departments of Medicine, Washington University School of Medicine, St. Louis, MO, USA; Emory University School of Medicine; Division of Virology, Institute of Medical Science, University of Tokyo; National Institute of Infectious Diseases; University of Tokyo, Institute of Medical Science; Influenza Research Institute, Department of Pathobiological Sciences, School of Veterinary Medicine, University of Wisconsin-Madison; National Center for Global Health and Medicine Research Institute; Department of Animal Dairy, and Veterinary Sciences, College of Agriculture and Applied Sciences, Utah State University; Institute of Medical Sciences, University of Tokyo; Department of Animal Dairy, and Veterinary Sciences, College of Agriculture and Applied Sciences, Utah State University; Department of Animal Dairy, and Veterinary Sciences, College of Agriculture and Applied Sciences, Utah State University; University of Wisconsin-Madison; Department of Pathobiological Sciences, School of Veterinary Medicine. University of Wisconsin, Madison; Universidad Nacional de Colombia; Wisconsin State Laboratory of Hygiene; Center for Childhood Infections and Vaccines of Children’s Healthcare of Atlanta, Department of Pediatrics, Emory Vaccine Center, Emory University School of Medicine; Wisconsin State Laboratory of Hygiene; Department of Microbiology and Immunology, University of Iowa.; Department of Microbiology and Immunology, University of Iowa.; Utah State University; Emory; Washington University; Washington University in St. Louis School of Medicine; The University of Tokyo; University Of Tokyo; University of Tokyo; University of Iowa; St. Jude Children’s Research Hospital; National Institutes of Health; Emory University School of Medicine; Icahn School of Medicine at Mount Sinai; Icahn School of Medicine at Mount Sinai; National Institute of Infectious Diseases; Washington University; University of Wisconsin-Madison; NIH; Vaccine Research Center, NIH; Vaccine Research Centre, NIH; VRC/NIAID/NIH; National Institutes of Health; Washington University School of Medicine; St. Jude Children’s Research Hospital, Memphis, TN, USA; St. Jude Children’s Research Hospital, Memphis, TN, USA; St Jude Children’s Research Hospital; Department of Infectious Diseases, St. Jude Children’s Research Hospital; Department of Infectious Diseases, St. Jude Children’s Research Hospital, Memphis, TN, USA; Department of Infectious Diseases, St. Jude Children’s Research Hospital; Icahn School of Medicine at Mount Sinai; Icahn School of Medicine at Mount Sinai; Icahn School of Medicine at Mount Sinai; Icahn School of Medicine at Mount Sinai; Icahn School of Medicine at Mount Sinai; Icahn School of Medicine at Mount Sinai

## Abstract

Despite the development and deployment of antibody and vaccine countermeasures, rapidly-spreading SARS-CoV-2 variants with mutations at key antigenic sites in the spike protein jeopardize their efficacy. The recent emergence of B.1.1.529, the Omicron variant1,2, which has more than 30 mutations in the spike protein, has raised concerns for escape from protection by vaccines and therapeutic antibodies. A key test for potential countermeasures against B.1.1.529 is their activity in pre-clinical rodent models of respiratory tract disease. Here, using the collaborative network of the SARS-CoV-2 Assessment of Viral Evolution (SAVE) program of the National Institute of Allergy and Infectious Diseases (NIAID), we evaluated the ability of multiple B.1.1.529 Omicron isolates to cause infection and disease in immunocompetent and human ACE2 (hACE2) expressing mice and hamsters. Despite modeling and binding data suggesting that B.1.1.529 spike can bind more avidly to murine ACE2, we observed attenuation of infection in 129, C57BL/6, and BALB/c mice as compared with previous SARS-CoV-2 variants, with limited weight loss and lower viral burden in the upper and lower respiratory tracts. Although K18-hACE2 transgenic mice sustained infection in the lungs, these animals did not lose weight. In wild-type and hACE2 transgenic hamsters, lung infection, clinical disease, and pathology with B.1.1.529 also were milder compared to historical isolates or other SARS-CoV-2 variants of concern. Overall, experiments from multiple independent laboratories of the SAVE/NIAID network with several different B.1.1.529 isolates demonstrate attenuated lung disease in rodents, which parallels preliminary human clinical data.

## Introduction

Severe acute respiratory syndrome coronavirus 2 (SARS-CoV-2) has caused the global Coronavirus Disease 2019 (COVID-19) pandemic resulting in millions of deaths worldwide. The extensive morbidity and mortality associated with the COVID-19 pandemic made the development of SARS-CoV-2 vaccines, antibody-based countermeasures, and direct acting antiviral agents a global health priority. As part of the development process, several key animal models of SARS-CoV-2 infection and lung pathogenesis were developed in mice, hamsters, nonhuman primates (NHP) and other animals for rapid testing and evaluation^3^. Remarkably, several highly effective vaccines and antibody therapeutics targeting SARS-CoV-2 spike protein gained regulatory approval and were deployed with hundreds of millions of doses given worldwide (https://covid19.who.int). While these measures markedly reduced numbers of infections, hospitalizations, and deaths, their efficacy has been jeopardized by emergence of highly transmissible variant SARS-CoV-2 strains with mutations in the spike protein that could compromise protective immune responses and therapeutics.

Currently available vaccines and antibody countermeasures were developed using the SARS-CoV-2 spike protein from strains circulating during the early phases of the pandemic in 2020. The SARS-CoV-2 spike protein engages angiotensin-converting enzyme 2 (ACE2) on the surface of human cells to facilitate entry and infection of cells^4^. Upon cell attachment, SARS-CoV-2 spike proteins are cleaved by host proteases into S1 and S2 fragments. The S1 protein includes the N-terminal (NTD) and receptor binding (RBD) domains, whereas the S2 protein promotes membrane fusion. The RBD, in particular, is the target of many potently neutralizing monoclonal^5–9^ (mAb) and serum polyclonal antibodies^10^. Although SARS-CoV-2 spike proteins from strains early in the pandemic bound to ACE2 from multiple susceptible animal species (*e.g*., hamster, ferret, and NHP), they did not bind mouse ACE2, which explained why conventional laboratory strains of mice could not be infected efficiently by SARS-CoV-2^4,11^; indeed, mice could become susceptible through ectopic expression of hACE2 via a transgene^12–14^, adenoviral vector delivery^15,16^, or expression of hACE2 by the mouse ACE2 promoter^17–19^. However, later in the pandemic, several variant strains (*e.g*., B.1.1.7 (Alpha), B.1.351 (Beta), B.1.1.28 (Gamma), and B.1.621 (Mu)) acquired a mouse-adapting spike mutation (N501Y), which allowed engagement of murine ACE2 and productive infection of laboratory strains of mice without ectopic hACE2 expression^20–22^.

In late November of 2021, the Omicron (B.1.1.529) variant emerged, which has the largest number (>30) of mutations, deletions, or insertions in the spike protein described to date. This number of changes in the spike has raised concerns for escape from protection by vaccines and therapeutic mAbs. Most B.1.1.529 isolates have a constellation of changes in the spike protein including: A67V, D69–70, T95I, G142D, D143–145, D211, L212I, insertion 214EPE, G339D, S371L, S373P, S375F, K417N, N440K, G446S, S477N, T478K, E484A, Q493R, G496S, Q498R, N501Y, Y505H, T547K, D614G, H655Y, N679K, P681H, N764K, D796Y, N856K, Q954H, N969K, and L981F. The presence of the N501Y mutation along with additional mutations (K417, E484, Q493, Q498, and N501) at sites associated with mouse adaptation by serial passage^23–28^ suggested that B.1.1.529 should infect laboratory strains of mice^29^. Indeed, one recent study speculated that because mutations in the B.1.1.529 spike protein overlap with ones known to promote adaptation to mouse hosts, the progenitor of B.1.1.529 jumped from humans to mice, and then back into humans^30^. In support of this, B.1.1.529 but not Wuhan-1 RBD binds to murine ACE2^31^. Lastly, hamsters have been a valuable pre-clinical animal model for assessing countermeasures against SARS-CoV2 and variants. Hamsters are highly susceptible to SARS-CoV-2 infection with similar pathological changes seen in lung tissues from COVID-19 patients^3,32,33^. Here, using experimental data from multiple laboratories of the SAVE/NIAID consortium, we report on the infectivity of multiple B.1.1.529 isolates in mice and hamsters, two key rodent models of SARS-CoV-2 infection and pathogenesis that are used to model human disease and evaluate countermeasures.

## Results

### B.1.1.529 infection in mice.

**Figure 1:**
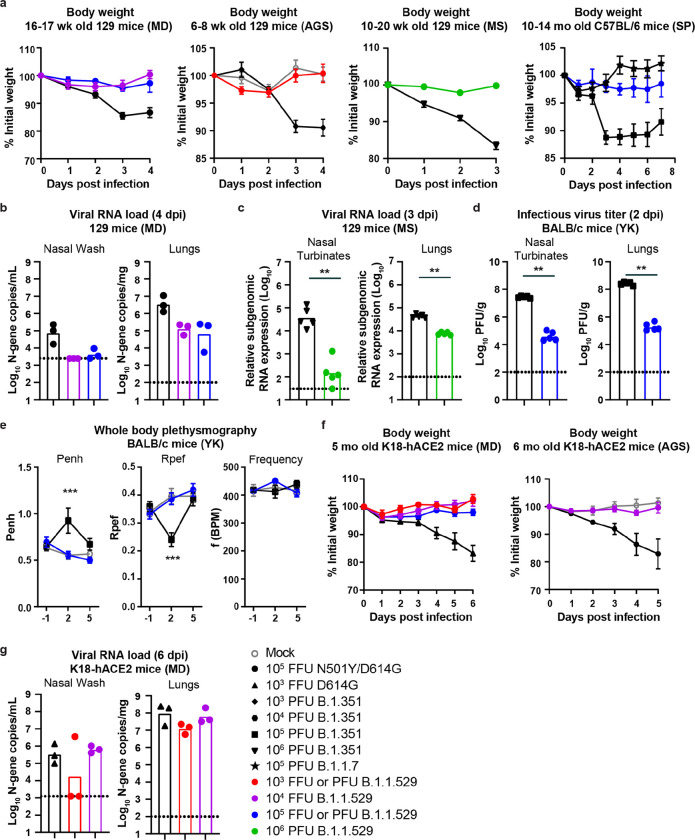
B.1.1.529 is attenuated in different mouse models of SARS-CoV-2 infection. **a,** *Far left:* Weight change in 129 mice inoculated via intranasal route with 10^4^ (purple circles, n = 6) or 10^5^ (blue circles, n = 6) FFU of B.1.1.529 (strain hCoV-19/USA/WI-WSLH-221686/2021) or 10^5^ FFU of WA1/2020 N501Y/D614G (black circles, n = 6). *Center left:* Weight change in mock-infected 129 mice (grey circles, n = 4) or 129 mice inoculated intranasally with 10^3^ (red circles, n = 5) PFU of B.1.1.529 (strain hCoV-19/USA/NY-MSHSPSP-PV44476/2021) or B.1.351 variant of SARS-CoV-2 (black diamonds, n = 3). *Center right:* Weight change in 129 mice inoculated intranasally with 10^6^ PFU of B.1.1.529 (strain hCoV19/EHC_C19_2811C, green circles, n = 5) or B.1.351 (black triangles, n = 5). *Far right:* Weight change in 10 to 14-month-old C57BL/6 mice inoculated intranasally with 10^5^ PFU of B.1.1.529 (strain hCoV19/EHC_C19_2811C, blue circles, n = 4), B.1.1.7 (black star, n = 10), or B.1.351 (black squares, n = 18). Data are mean ± SEM. **b,** Nasal wash and lung viral RNA levels in 129 mice inoculated with 10^4^ (purple circles, n = 3) or 10^5^ (blue circles, n = 3) FFU of B.1.1.529 (strain hCoV-19/USA/WI-WSLH-221686/2021) or 10^5^ FFU of WA1/2020 N501Y/D614G (black circles, n = 3). **c,** Nasal turbinates and lung viral RNA levels in 129 mice inoculated with 10^6^ PFU of B.1.1.529 (strain hCoV19/EHC_C19_2811C, green circles, n = 5) or B.1.351 (black triangles, n = 5) (** *P* < 0.01, by Mann-Whitney U test). **d,** Nasal turbinates and lung virus titers from BALB/c mice inoculated with 10^5^ PFU of B.1.1.529 (strain hCoV-19/Japan/NC928–2N/2021, blue circles, n = 5) or B.1.351 (black squares, n = 5) (** *P* < 0.001, by Mann-Whitney U test). **e,** Pulmonary function analysis in infected BALB/c mice. Penh, a surrogate marker for bronchoconstriction or airway obstruction, was measured by whole body plethysmography. Data are presented as the mean ± SEM. P values were calculated by using pairwise comparisons after a linear mixed model analysis (***P < 0.001). Asterisks indicate statistically significant differences between B.1.351-infected (n = 5) and B.1.1.529-infected (n = 5) or uninfected animals (n = 5). **f,** *Left:* Weight change in 5-month-old K18-hACE2 transgenic mice inoculated intranasally with 10^3^ (red circles, n = 3), 10^4^ (purple circles, n = 6), or 10^5^ (blue circles, n = 3) FFU of B.1.1.529 (strain hCoV-19/USA/WI-WSLH-221686/2021) or 10^3^ FFU of WA1/2020 D614G (black triangles, n = 6). *Right:* Weight change in 6-month-old K18-hACE2 transgenic mice inoculated intranasally with 10^4^ (purple circles, n = 6) PFU of B.1.1.529 (strain hCoV-19/USA/NY-MSHSPSP-PV44476/2021), or 10^4^ PFU of B.1.351 (black hexagon, n = 6)). Age-matched uninfected mice (grey circles, n = 4) were included as controls. Data are presented as the mean ± SEM. **g,** Nasal wash and lung viral RNA levels in K18-hACE2 mice inoculated with 10^3^ FFU of WA1/2020 D614G (black triangles, n = 3), or 10^3^ (red circles, n =3) or 10^4^ (purple circles, n =3) FFU of B.1.1.529 (strain hCoV-19/USA/WI-WSLH-221686/2021). The results are from one experiment and each symbol in **b, c, d,** and **g** represents an individual animal. The dotted line is the limit of detection. Infection studies in panels **a, b** and **f** with WA1/2020 N501Y/D614G are shown as comparisons and were adapted from published data^22^.

Based on the presence of several mutations that are considered mouse-adapting, we predicted that B.1.1.529 might productively infect immunocompetent mice and cause lung disease as seen with other recombinant strains (WA1/2020 N501Y/D614G) or variants (e.g., B.1.351) containing N501Y mutations. We first tested B.1.1.529 in 129 mice. Three laboratories in the SAVE/NIAID consortia (M.S.D. [St. Louis], M.S.S. [Atlanta], and A.G.S. [New York]) independently inoculated 6 to 8-week-old (A.G.S.) or 10 to 20-week-old (M.S.D and M.S.S.) 129 mice with 10^4^, 10^5^ or 10^6^ infectious units (plaque- [PFU] or focus-forming [FFU] units) of three different B.1.1.529 strains (isolated in Wisconsin, Georgia, and New York). At 3 to 4 days-post-infection (dpi) with SARS-CoV-2 strains encoding N501Y substitution (e.g. WA1/2020 N501Y/D614G or B.1.351), 129 mice sustain 10 to 15% loss of body weight. However, after inoculation of B.1.1.529, 129 mice failed to lose weight in all three laboratories (Fig 1a). Similarly, aged (10 to 14-month-old) C57BL/6 breeders also did not lose significant weight after B.1.1.529 infection, whereas those infected with B.1.351 did (Fig 1a).

We next measured the viral burden in the upper and lower respiratory tract of B.1.1.529-infected 129 mice and compared this to side-by-side infection with B.1.351 or historical data with WA1/2020 N501Y/D614G^22,34^. At 3 or 4 dpi, 129 mice infected with WA1/2020 N501Y/D614G or B.1.351 sustained high levels of infection in the nasal wash, nasal turbinates, and lungs (Fig 1b–c); this level of infection previously was associated with histological evidence of mild pneumonia^20,22,34^. At 3 dpi, B.1.1.529 infection in the nasal turbinates and lungs was 10 to 100-fold lower than in B.1.351-infected mice (*P* < 0.01, Fig 1c). At 4 dpi, in contrast to WA1/2020 N501Y/D614G infection, we were unable to detect B.1.1.529 viral RNA in the nasal wash. Moreover, at this time point, 10 to 100-fold lower levels of viral RNA were present in lung homogenates of B.1.1.529-infected mice compared to WA1/2020 N501Y/D614G-infected mice (Fig 1b). Based on weight change and viral burden, B.1.1.529 appeared attenuated in 129 mice.

Members of the SAVE/NIAID group (Y.K.) also tested B.1.1.529 in BALB/c mice and compared this to infection with B.1.351 variant. At 2 dpi, infectious virus levels in the nasal turbinates and lungs were significantly lower (~1,000-fold, *P* < 0.001) in BALB/c mice infected with B.1.1.529 compared to B.1.351 virus (Fig 1d). We next used a whole-body plethysmography system^35^ to measure pulmonary function in infected BALB/c mice. At 2 dpi, whereas B.1.351 caused an increase (*P* < 0.001) in the lung enhanced pause (Penh), a surrogate marker for bronchoconstriction or airway obstruction, B.1.1.529 did not (Fig 1e). Consistent with this result, the ratio of peak expiratory flow (Rpef) was decreased at 2 dpi in BALB/c mice infected with B.1.351 but not B.1.1.529 (*P* < 0.001, Fig 1e). Thus, based on multiple parameters, lung infection and disease after B.1.1.529 was attenuated compared to other strains.

Given the attenuation in several strains of conventional laboratory mice, two groups tested B.1.1.529 infection in 5 to 6-month-old (M.S.D. and A.G.S.) K18-hACE2 transgenic mice, which express hACE2 under an epithelial cytokeratin promoter^12^, and are more susceptible to SARS-CoV-2 infection and disease^14^. At intranasal inoculating doses ranging from 10^3^ to 10^5^ infectious units of B.1.1.529, weight loss was not observed over the first 5 to 6 days of infection in younger or older K18-hACE2 mice (Fig 1f). These data contrasts with historical results with WA1/2020 D614G or variant (e.g., B.1.351) SARS-CoV-2 strains^14,22,34,36^, which uniformly induce weight loss starting at 4 dpi. Although we observed clinical attenuation of B.1.1.529 in K18-hACE2 mice, the virus nonetheless accumulated in the upper and lower respiratory tract (Fig 1g).

### B.1.1.529 infection in hamsters.

**Figure 2:**
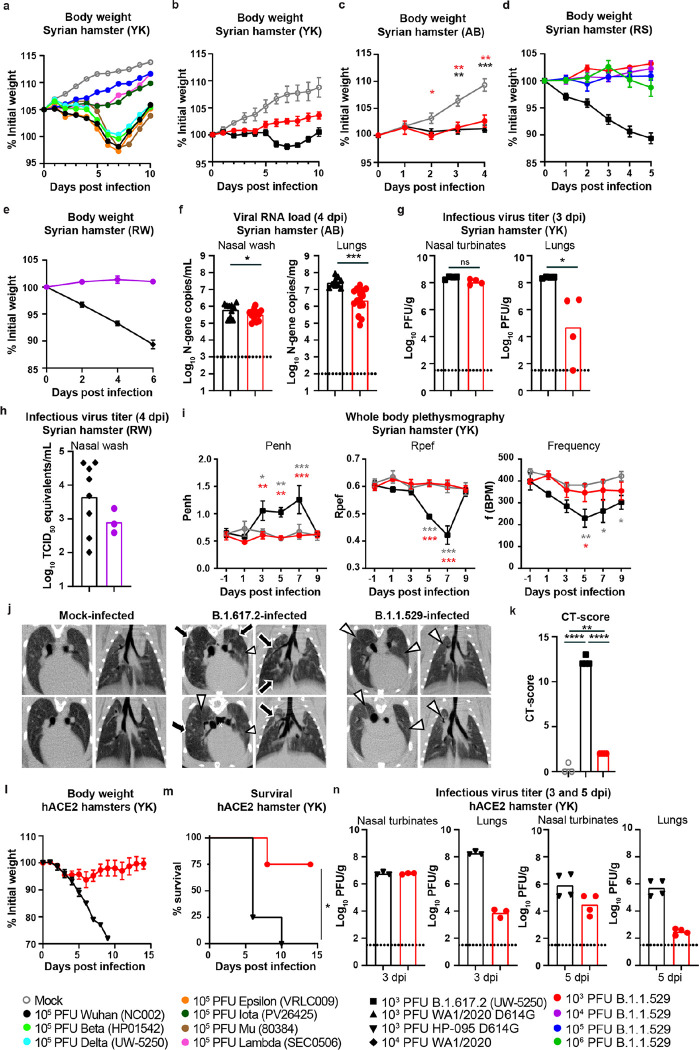
B.1.1.529 is attenuated in wild-type and human ACE2-transgenic Syrian hamsters. **a,** Weight change in uninfected age-matched Syrian hamsters (open grey circles, n = 4) or in hamsters inoculated intranasally with 10^5^ PFU (n = 9) of B.1.1.529 (strain hCoV-19/Japan/NC928–2N/2021, blue circles), B.1.351 (Beta, HP01542), B.1.617.2 (Delta, strain hCoV-19/USA/WI-UW-5250/2021), B.1.621 (Mu, 80384), B.1.427 (Epsilon, VRLC009), B.1.526 (Iota, PV26425), C.37 (Lambda, SEC0506), or Wuhan-1 (black circles). **b,** Weight change in uninfected age-matched Syrian hamsters (open grey circles, n = 3) or in hamsters inoculated intranasally with 10^3^ PFU of B.1.1.529 (strain hCoV-19/Japan/NC928–2N/2021, red circles, n = 4) or B.1.617.2 (black squares, n = 4). Data are mean ± SEM. c, Weight change in uninfected age-matched Syrian hamsters (open grey circles, n = 9) or in hamsters inoculated intranasally with 10^3^ PFU of B.1.1.529 (strain hCoV-19/USA/WI-WSLH-221686/2021, red circles, n = 10) or WA1/2020 D614G (black triangles, n = 6). Data are mean ± SEM. (* *P* < 0.05, ** *P* < 0.01, *** *P* < 0.001 by two-way ANOVA with a Dunnett’s correction). **d,** Weight change in Syrian hamsters inoculated intranasally with 10^3^ (red circles, n = 4), 10^4^ (purple circles, n = 4), 10^5^ (blue circles, n = 4), or 10^6^ (green circles, n = 4) PFU of B.1.1.529 (strain hCoV19/EHC_C19_2811C) or 10^3^ PFU of B.1.617.2 (black squares, n = 4). Data are mean ± SEM. **e,** Weight change in hamsters inoculated intranasally with 10^4^ PFU of B.1.1.529 (strain hCoV19/EHC_C19_2811C, purple circles, n = 5) or WA1/2020 (black diamonds, n = 9). Data are mean ± SEM. **f,** Nasal wash and lung viral RNA load in wild-type Syrian hamsters inoculated with 10^3^ PFU of WA1/2020 D614G (black triangles, n = 15) or B.1.1.529 (strain hCoV-19/USA/WI-WSLH-221686/2021, red circles, n = 15) (*** *P* < 0.001, * *P* < 0.05 by unpaired t-test). **g,** Nasal turbinates and lung virus titer in wild-type Syrian hamsters inoculated with 10^3^ PFU of B.1.617.2 (strain hCoV-19/USA/WI-UW-5250/2021, black squares, n = 4) or B.1.1.529 (strain hCoV-19/Japan/NC928–2N/2021, red circles, n = 4) (* *P* < 0.05, ns = not significant; Mann Whitney U test). **h**, Nasal wash viral RNA load (TCID_50_ equivalents/mL) in wild-type Syrian hamsters inoculated with 10^4^ PFU of WA1/2020 (black diamonds, n = 8) or B.1.1.529 (strain hCoV19/EHC_C19_2811C, purple circles, n = 3). **i,** Pulmonary function analysis in infected Syrian hamsters. Penh was measured by whole body plethysmography. Data are mean ± SEM. P values were calculated by using pairwise comparisons after a linear mixed model analysis (*** *P* < 0.001, ** *P* < 0.01, * *P* < 0.05). Asterisks indicate statistically significant differences between B.1.617.2-infected (n = 4) and B.1.1.529-infected (n = 4) or uninfected animals (n = 3). j, Representative micro-CT axial and coronal images of the lungs of mock-infected (n = 3) or B.1.617.2- (n = 4) and B.1.1.529-infected (n = 4) hamsters on 7 dpi. Lung abnormalities included multifocal nodules (black arrows), ground glass opacity (white arrowheads), and regions of lung consolidation (white arrows) that were peripheral, bilateral, and multilobar. Pneumomediastinum is indicated with white asterisks. **k,** CT score for uninfected hamsters (open grey circles, n = 3) or those inoculated with 10^3^ PFU of B.1.617.2 (black squares, n = 4) or B.1.1.529 (strain hCoV-19/Japan/NC928–2N/2021, red circles, n = 4). **l,** Weight change in hACE2-transgenic Syrian hamsters inoculated intranasally with 10^3^ PFU of HP-095 D614G (black triangles, n = 4) or B.1.1.529 (strain hCoV-19/USA/WI-WSLH-221686/2021, red circles, n = 4). **m**, Survival analysis of hACE2-transgenic Syrian hamsters after inoculation with 10^3^ PFU of HP-095 D614G (black triangles, n = 4) or B.1.1.529 (strain hCoV-19/USA/WI-WSLH-221686/2021, red circles, n = 4) (* *P* < 0.05; log-rank test). **n**, Nasal turbinate and lung infectious virus titer by plaque assay at 3 and 5 dpi from hACE2-transgenic Syrian hamsters inoculated with 10^3^ PFU of HP-095 D614G (black triangles) or B.1.1.529 (strain hCoV19/USA/WI-WSLH-221686/2021, red circles); n = 3 (3 dpi), n = 4 (5 dpi). The results in the Figure are from one (**a, b, d-e,** and **g-n**) or two to three independent (**c,** and **f**) experiments. Each symbol represents an individual animal. Dotted lines represent the limit of detection.

Because Syrian hamsters are a pivotal model for studying SARS-CoV-2 pathogenesis and evaluating countermeasures^3^, four members of our SAVE/NIAID team (R.A.S., A.C.M.B, R.J.W., and Y.K.) tested three different B.1.1.529 strains (from Wisconsin, Georgia, and Japan) for their ability to infect and cause disease. Whereas intranasal infection with historical or other variant SARS-CoV-2 strains generally resulted in ~10 to 15% reduction in body weight over the first week, we observed no weight loss in any of the hamsters inoculated with B.1.1.529, regardless of inoculating dose (Fig 2a–e). However, animals infected with B.1.1.529 did not gain body weight as rapidly as uninfected hamsters. Viral RNA analysis at 4 dpi showed lower levels of B.1.1.529 infection in the nasal wash (2-fold, *P* < 0.05) and lungs (12-fold, *P* < 0.001) compared to WA1/2020 D614G ([A.C.M.B.]; Fig 2f). A comparison of infectious viral burden in tissues at 3 dpi between B.1.617.2 (Delta) and B.1.1.529 (Omicron) viruses [Y.K.] showed virtually no difference in nasal turbinates but substantially less infection of B.1.1.529 in the lungs of most animals (Fig 2g). A comparison of viral RNA levels between WA1/2020 and B.1.1.529 viruses (R.J.W) in nasal washes at 4 dpi did not show substantial differences in nasal wash titers (Fig 2h). Thus, in hamsters, upper, but not lower, respiratory tract infection by B.1.1.529 appears relatively intact.

We also used whole-body plethysmography to measure pulmonary function in infected Syrian hamsters (Y.K.). Starting at 3 dpi and continuing until 7 dpi, infection with B.1.617.2 caused an increase (*P* < 0.05) in the lung enhanced pause (Penh), whereas B.1.1.529 infection did not (Fig 2i). Consistent with this result, the ratio of peak expiratory flow (Rpef) was decreased at 5 and 7 dpi in animals infected with B.1.617.2 but not B.1.1.529 (*P* < 0.001, Fig 2i). Finally, hamsters infected with B.1.617.2, but not B.1.1.529, demonstrated a decrease in respiratory rate compared to uninfected control animals. Thus, based on multiple functional parameters, lung infection and disease after B.1.1.529 infection was attenuated compared to other variant strains.

We next performed microcomputed tomography (micro-CT) to assess for lung abnormalities in hamsters at 7 dpi. We used a previously defined CT severity score (see Legend and Methods) to evaluate animals for nodules, ground glass opacities, and regions of lung consolidation^35^. Micro-CT analysis revealed lung abnormalities in all B.1.617.2- infected hamsters on 7 dpi that were consistent with commonly reported imaging features of COVID-19 pneumonia^37^. In comparison, analysis of B.1.1.529-infected hamsters on 7 dpi revealed patchy, ill-defined ground glass opacity consistent with minimal to mild pneumonia. Accordingly, Syrian hamsters infected with B.1.617.2 had a high CT disease score (~12), whereas those infected with B.1.1.529 had a substantially lower disease score (~2) that was only slightly greater than mock-infected hamsters (Fig 2j–k).

**Figure 3:**
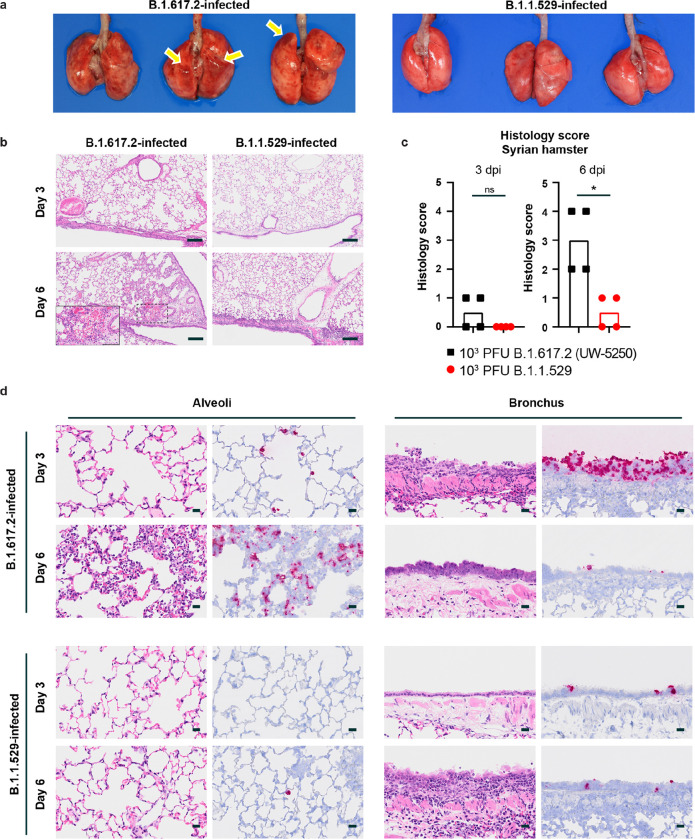
Pathological findings in the lungs of SARS-CoV-2 infected Syrian hamsters. The hamsters were infected with B.1.617.2 or B.1.1.529 variant of SARS-CoV-2 and sacrificed on 3 days (n = 4) and 6 days (n = 4) post infection for histopathological examinations. **a,** Macroscopic images of the lungs obtained from B.1.617.2 or B.1.1.529-infected Syrian hamsters at 6 dpi. Yellow arrows indicate areas of pulmonary hemorrhage. **b,** Representative histopathological images for the lung sections obtained from the animals infected with B.1.617.2 or B.1.1.529 at 3 or 6 dpi are shown at low magnification. Scale bars, 200 μm. Focal alveolar hemorrhage found in B.1.617.2-infected animals at 6 dpi is highlighted by dashed lines and shown at higher magnification in the inset image (scale bar indicating 100 μm). **c,** Histopathological score of viral pneumonia in the lungs of infected hamsters. Score was determined based on the percentage of alveolitis in a given section collected from each animal in each group using the following scoring: 0, no pathological change; 1, affected area (≤10%); 2, affected area (<50%, >10%); 3, affected area (≥50%); an additional point was added when pulmonary edema and/or alveolar hemorrhage was observed. Data are expressed as scatter plots with the median score ± 95% confidential interval. Each dot represents the score of each animal. Comparison between B.1.617.2 (n = 4) or B.1.1.529 (n = 4) was performed using Kruskal-Wallis test with a Dunn’s post-tests. ns, not significant; *, *P* < 0.05. **d,** RNA *in situ* hybridization (RNA-ISH) for SARS-CoV-2 viral RNA. Representative RNA-ISH images for the alveoli and bronchi of hamsters infected with B.1.617.2 (n = 4) or B.1.1.529 (n = 4) virus at 3 or 6 dpi are shown. Left panels, alveolar region. Right panels, bronchial region. Scale bars, 20 μm.

We also compared lung pathology in Syrian hamsters after infection with B.1.617.2 or B.1.1.529 viruses. Macroscopically, the lungs obtained from the B.1.617.2-infected hamsters showed congestion and/or hemorrhage, but this was absent in B.1.1.529-infected animals (Fig. 3a). At the microscopic level, immune cell infiltration and inflammation were present in the peribronchial regions of the lungs at 3 dpi with B.1.617.2. Moreover, at 6 days post B.1.617.2 infection, extensive infiltration of neutrophils and lymphocytes in the alveolar space and walls was accompanied by focal pulmonary edema and alveolar hemorrhage (Fig 3b, inset), and regenerative changes in the bronchial epithelia became prominent (Fig 3b). In contrast, in B.1.1.529-infected hamsters, small foci of inflammation in the alveoli and peribronchial regions were observed only at 6 dpi (Fig 3b). A semi-quantitative histopathology severity score of viral pneumonia at 6 dpi showed a worse score after B.1.617.2 than B.1.1.529 infection (Fig 3c). Consistent with these findings, after B.1.617.2 infection, viral RNA was detected readily in the alveoli and bronchial epithelia in lung tissue sections at 3 and 6 dpi (Fig 3d). In comparison, after B.1.1.529 infection, fewer bronchial epithelial cells and alveoli were positive for viral RNA at either time point (Fig 3d). Collectively, these findings suggest that the B.1.1.529 Omicron variant replicates less efficiently in the lungs of Syrian hamsters, which results in less severe pneumonia compared to the B.1.617.2 Delta variant.

Although hamsters are susceptible to SARS-CoV-2 infection without a requirement for host adaptation and show some similarities to that observed in COVID-19 patients, they develop self-limiting clinical and respiratory disease. Even though hamster ACE2 can serve as a receptor for SARS-CoV-2 spike protein, some of the contact residues in human ACE2 are not conserved^38^, which could diminish infectivity. Moreover, ACE2 expression levels on particular cells in the respiratory tract may differ slightly between hamsters and humans, which could impact infectivity and clinical outcome. To develop a more susceptible hamster model, members of the consortium (Y.K.) used transgenic hamsters (generated by Z.W.) expressing human ACE2 under the epithelial cytokeratin-18 promoter^39^. Whereas intranasal inoculation of 10^3^ PFU of HP-095 D614G virus resulted in marked weight loss within the first week (Fig 2l) and uniform mortality by 10 dpi (Fig 2m), less weight loss and death (*P* < 0.05) were observed after infection with 10^3^ PFU of B.1.1.529. Consistent with these clinical data, 1,000 to 10,000-fold lower levels of infectious virus were measured in the lungs of hACE2 transgenic hamsters challenged with B.1.1.529 compared to the HP-095 D614G virus at 3 and 5 dpi (Fig 2n). Notably, and as seen in wild-type Syrian hamsters, smaller differences in infection were observed in the nasal turbinates. Thus, B.1.1.529 infection in the lung is attenuated in both wild-type and hACE2 transgenic hamsters.

## Discussion

Our experiments suggest that compared to other SARS-CoV-2 isolates (e.g., B.1.351 or B.1.617.2), the B.1.1.529 Omicron variant infection is attenuated in laboratory mice and hamsters for causing infection and/or disease. While these results are consistent with the very preliminary clinical data in humans suggesting that B.1.1.529 causes a more transmissible yet possibly milder respiratory infection^40,41^, the basis for the attenuation in rodents remains unknown. One pre-print study suggests that B.1.1.529 replicates faster in the human bronchus and less in lung cells, which may explain its greater transmissibility and putative lower disease severity^42^; although it remains unclear if these observations extend to rodents, we observed less infection of hamster bronchial cells *in vivo* with B.1.1.529 Omicron than B.1.617.2 Delta virus. We also measured lower viral burden in nasal washes and turbinates in 129 mice compared to other SARS-CoV-2 strains. The attenuation in mice was unexpected given that B.1.1.529 has multiple mutations in the RBD that are sites (K417, E484, Q493, Q498, and N501) associated with adaptation for mice^23–25^. Moreover, the attenuation in hamsters also was surprising, given that all prior SARS-CoV-2 variants have replicated relatively equivalently and to high levels in this animal^35,43,44^. However, our results showing attenuation of B.1.1.529 in hamsters are consistent with another preliminary report^45^. Whereas the more than 30 substitutions (mutations, deletions, and insertions) in the B.1.1.529 could impact receptor engagement and cell entry, sequence changes in other structural, non-structural, and immune evasion proteins could affects replication, temperature sensitivity, cell-to-cell spread, cell and tissue tropism, dissemination, and induction of pro-inflammatory immune responses in a species-specific manner. Thus, detailed genetic and functional studies are required to define the basis of virological and clinical attenuation of B.1.1.529 in mice and hamsters.

Although we observed clinical attenuation of B.1.1.529 in mice and hamsters, these animals likely will still have utility in evaluating vaccine, antibody, or small molecule inhibitors. All of the mice and hamsters tested, to varying degrees, showed evidence of viral replication and dissemination to the lower respiratory tract, which could be prevented or mitigated by prophylactic or therapeutic countermeasures. The most severe B.1.1.529 infection and disease was observed in hACE2 expressing mice and hamsters, which is consistent with results with other SARS-CoV-2 strains and variants^14,22,39,46^, and possibly related to the enhanced interactions between hACE2 and the B.1.1.529 spike protein^47^.

These *in vivo* studies were performed as part of the SAVE/NIAID consortium and reflect a collaborative network of expert investigators that communicates multiple times per week to expedite progress on emerging SARS-CoV-2 variants that threaten vaccine efficacy. Several key advantages with relevance to small animal models arise from this interactive format: (a) Animal experiments were reproduced across laboratories providing immediate confidence in results and interpretation; (b) Multiple independent B.1.1.529 isolates were used limiting the possibility of skewing of particular sequence adaptations in a particular strain from one laboratory that could impact results; (c) Multiple strains and vendors of mice (129, BALB/c, C57BL/6, and K18-hACE2) and hamsters (Syrian Golden and hACE2 transgenic) at different ages were tested in a short time span allowing for the accumulation of a robust and larger data set and an appreciation of the generality of the results; and (d) The groups used an overlapping set of metrics (weight measurements, viral load, plethysmography, histopathology, and micro-CT imaging) to evaluate and compare infection and disease in the different animal models.

Nonetheless, we note several limitations to the conclusions of our study: (1) While our experiments, showing attenuation of B.1.1.529 in mice and hamsters, correlate with preliminary clinical data in humans, further experiments in NHPs and evaluation of human data are needed to corroborate these findings. Moreover, all of our studies ended at relatively early time points to allow for acquisition of respiratory tract tissues and titration of virus. It is possible that B.1.1.529 has altered tropism for other organs in rodents or requires additional time for replication and dissemination; (2) In all studies, we used the prevailing B.1.1.529 Omicron isolate that lacks an R346K mutation. While ~8% of B.1.1.529 sequences in GISAID currently have an R346K mutation, this substitution or others in genes apart from spike might further affect virulence in rodents. Although one of the B.1.1.529 isolates we tested (A.G.S., hCoV-19/USA/NY-MSHSPSP-PV44476/2021) contains an additional A701V mutation in spike near the furin cleavage site, it was still attenuated in mice compared to the B.1.351 virus; (3) Our data are focused on studying infection in a given animal and do not address other key questions in the field including vaccine protection, transmission, or antibody or small molecule drug protection; and (4) Due to time constraints, detailed pathological analyses were not performed for all of the animal species studied.

In summary, our studies by a network of investigators at multiple sites with several independently isolated B.1.1.529 strains rapidly and reproducibly demonstrated attenuated infection in multiple strains of laboratory mice and hamsters. Despite the attenuation of B.1.1.529 Omicron variants in causing infection in rodents, some of these pre-clinical models likely will retain utility for testing existing and novel vaccines and therapeutics given that viral replication occurs and some inflammatory and clinical disease is observed. Studies are ongoing to determine the basis for attenuation in mice and hamsters and to determine how this relates to the patterns of B.1.1.529 Omicron infection seen in humans.

## Methods

### Cells.

Vero-TMPRSS2^35,48,49^ and Vero-hACE2-TMPRSS2^50^ cells were cultured at 37°C in Dulbecco’s Modified Eagle medium (DMEM) supplemented with 10% fetal bovine serum (FBS), 10 mM HEPES pH 7.3, and 100 U/ml of penicillin–streptomycin. Vero-TMPRSS2 cells were supplemented with 5 mg/mL of blasticidin or 1 mg/mL of geneticin (depending on the cell line) and in some cultures with plasmocin. Vero-hACE2-TMPRSS2 cells were supplemented with 10 μg/mL of puromycin. All cells routinely tested negative for mycoplasma using a PCR-based assay.

### Viruses.

The WA1/2020 recombinant strains with substitutions (D614G and/or N501Y/D614G) were described previously^51^. The B.1.1.529 isolates (hCoV-19/USA/WI-WSLH-221686/2021 [Y.K., M.S.D., R.A.S., A.C.M.B., GISAID: EPI_ISL_7263803], hCoV-19/Japan/NC928–2N/2021 (NC928) [Y.K., GISAID: EPI_ISL_7507055], hCoV-19/USA/NY-MSHSPSP-PV44476/2021 [A.G.S., GISAID: EPI_ISL_7908052], and hCoV19/EHC_C19_2811C [M.S.S., S.P., and R.J.W. GISAID: EPI_ISL_7171744]) were obtained from nasal swabs and passaged on Vero-TMPRSS2 cells as described^33,35,49^. Other viruses used included: SARS-CoV-2/UT-NC002–1T/Human/2020/Tokyo (NC002^33^), SARS-CoV-2/UT-HP095–1N/Human/2020/Tokyo (HP-095; D614G), hCoV-19/USA/CA_CDC_5574/2020 (Alpha, B.1.1.7; BEI NR54011), hCoV-19/USA/MD-HP01542/2021 (Beta, B.1.351; HP01542), 20H/501Y.V2 (Beta, B.1.351), hCoV-19/USA/WI-UW-5250/2021 (Delta, B.1.617.2; UW-5250)^52^, hCoV-19/USA/CA-VRLC009/2021 (Epsilon, B.1.427; VRLC009), hCoV-19/USA/NYCPV26425/2021 (B.1.526, Iota; PV26425), hCoV-19/USA/217–80384/2021 (B.1.621, Mu; 80384), and hCoV-19/Colombia/SEC0506/2021 (C.37, Lambda; SEC0506). All viruses were subjected to next-generation sequencing as described^53^ to confirm the stability of substitutions and avoid introduction of adventitious mutations. All virus experiments were performed in an approved biosafety level 3 (BSL-3) facility.

### Animal experiments and approvals.

Animal studies were carried out in accordance with the recommendations in the Guide for the Care and Use of Laboratory Animals of the National Institutes of Health. The protocols were approved by the Institutional Animal Care and Use Committee at the Washington University School of Medicine (assurance number A3381–01), University of Wisconsin, Madison (V006426), St. Jude Children’s Research Hospital (Assurance number D16–00043), Emory University, University of Iowa (assurance number A3021–01), Icahn School of Medicine at Mount Sinai (PROTO202100007), BIOQUAL, Inc., and the Animal Experiment Committee of the Institute of Medical Science, the University of Tokyo (approval numbers PA19–72 and PA19–75). Virus inoculations were performed under anesthesia that was induced and maintained with ketamine hydrochloride and xylazine, and all efforts were made to minimize animal suffering.

### Mouse infection experiments.

Heterozygous K18-hACE2 C57BL/6J mice (strain 2B6.Cg-Tg(K18-ACE2)2Prlmn/J), 129 mice (strain: 129S2/SvPasCrl or 129S1/SvImJ), and C57BL/6 (strain 000664) mice were obtained from The Jackson Laboratory and Charles River Laboratories. BALB/c mice were purchased from Japan SLC Inc. Animals were housed in groups and fed standard chow diets. Infection experiments were performed as follows: (a) [M.S.D] 16- to 17-week-old female 129S2 mice were administered 10^4^ or 10^5^ FFU of B.1.1.529 [hCoV-19/USA/WI-WSLH-221686/2021] or other SARS-CoV-2 strains by intranasal administration; 5-month-old female K18-hACE2 mice were inoculated by intranasal route with 10^3^, 10^4^ or 10^5^ FFU of SARS-CoV-2. (b) [M.S.S.] 129S1 male and female mice were used were between 10–20 weeks of age. Mice were anesthetized with isoflurane and inoculated intranasally with virus (50 mL, 10^6^ PFU/mouse); (c) [Y.K.] Six-week-old female BALB/c mice were inoculated intranasally with 10^5^ PFU of hCoV-19/Japan/NC928–2N/2021 or hCoV-19/USA/MD-HP01542/2021; (d) [S.P.] Retired breeder female C57BL/6 mice (10 to 14-month-old) were anesthetized with ketamine-xylazine and inoculated intranasally with SARS-CoV-2 in a total volume of 50 μL of DMEM. Animal weight and health were monitored daily; and (e) [A.G.S.] 10- or 31-week-old female 129S1 mice and 6-month-old female K18-hACE2 mice were inoculated by intranasal route under light ketamine/xylazine sedation with 10^4^ PFU of hCoV-19/USA/NY-MSHSPSP-PV44476/2021 in a total volume of 50 μL.

### Hamster infection experiments.

Five-to-six-week-old male hamsters were obtained from Charles River Laboratories, Envigo, or Japan SLC Inc. The K18-hACE2 transgenic hamster line was developed with a piggyBac-mediated transgenic approach, in which the K18-hACE2 cassette from the pK18-hACE2 plasmid^12^ was transferred into a piggyBac vector, pmhyGENIE-3^54^, for pronuclear injection. hACE2 transgenic hamsters will be described in detail elsewhere^39^. Twelve-month-old transgenic female animals were used. Infection experiments were performed as follows: (a) [A.C.M.B] animals were challenged via intranasal route with 10^3^ PFU of WA1/2020 D614G or B.1.1.529 variant in 100 μL; (b) [Y.K.] Under *isoflurane* anesthesia, wild-type Syrian hamsters were intranasally inoculated with 10^3^ PFU or with 10^5^ PFU of SARS-CoV-2 strains in 30 μL. Body weight was monitored daily. For virological and pathological examinations, four hamsters per group were euthanized 3 and 6 dpi, and nasal turbinates and lungs were collected. The virus titers in the nasal turbinates and lungs were determined by plaque assays on Vero-TMPRSS2 cells. Human ACE2 transgenic hamsters were intranasally inoculated with 10^3^ PFU of HP-095 D614G or B.1.1.529 [hCoV-19/USA/WI-WSLH-221686/2021] in 50 μL. Body weight and survival were monitored daily, and nasal turbinates and lungs were collected at 3 and 5 dpi for virological analysis; (c) [R.A.S.] Six-week-old male Syrian golden hamsters were randomized into groups of 4 to 6 and inoculated with SARS-CoV-2 via delivery of 100 μL of appropriately diluted virus in PBS equally split between both nostrils. Weight change and clinical observations were collected daily; and (d) [R.J.W] while under *isoflurane* anesthesia, male 8–10 week old hamsters were inoculated intranasally with 10^4^ PFU of WA1/2020 or B.1.1.529 in 100 μL volume. Body weight and survival were monitored daily. Nasal washes were taken at 4 dpi for virological analysis.

### Measurement of viral burden.

#### (a) Mouse studies.

[M.S.D.] Tissues were weighed and homogenized with zirconia beads in a MagNA Lyser instrument (Roche Life Science) in 1000 μL of DMEM medium supplemented with 2% heat-inactivated FBS. Tissue homogenates were clarified by centrifugation at 10,000 rpm for 5 min and stored at −80°C. RNA was extracted using the MagMax mirVana Total RNA isolation kit (Thermo Fisher Scientific) on the Kingfisher Flex extraction robot (Thermo Fisher Scientific). Viral RNA (*N* gene) was reverse transcribed and amplified using the TaqMan RNA-to-CT 1-Step Kit (Thermo Fisher Scientific), and data were analyzed and normalized as described previously^55^. [Y.K.] The viral titers in the nasal turbinates and lungs were determined by plaque assay on Vero-TMPRSS2 cells as previously published^49^. [M.S.S.] At the indicated day post-infection, mice were euthanized with isoflurane overdose and one lobe of lung tissue was collected in an Omni Bead ruptor tube filled with Tri Reagent (Zymo, #R2050–1-200). Tissue was homogenized using an Omni Bead Ruptor 24 (5.15 ms, 15 seconds), then centrifuged to remove debris. RNA was extracted using a Direct-zol RNA MiniPrep Kit (Zymo, # R2051), then converted to cDNA using a High-capacity Reverse Transcriptase cDNA Kit (Thermo, #4368813). SARS-CoV-2 RNA-dependent RNA polymerase and subgenomic RNA were measured as described^27,56^. [M.S.S.] The subgenomic SARS-CoV-2 RNA levels were quantified in nasal turbinates and lungs by qRT-PCR as previously published^27,53^.

#### (b) Hamster studies.

[A.C.M.B.] Lungs were collected 4 dpi and homogenized in 1.0 mL DMEM, clarified by centrifugation (1,000 × g for 5 min) and stored at −80°C. Nasal washes were clarified by centrifugation (2,000 × g for 10 min) and the supernatant was stored at −80°C. To quantify viral load in lung tissue homogenates and nasal washes, RNA was extracted from 100 μL samples using E.Z.N.A.® Total RNA Kit I (Omega) and eluted with 50 μL of water. Four microliters RNA was used for real-time RT-qPCR to detect and quantify *N* gene of SARS-CoV-2 using TaqMan™ RNA-to-CT 1-Step Kit (Thermo Fisher Scientific) as described^57^. [Y.K.] The virus titers in the nasal turbinates and lungs were determined by plaque assay on Vero E6 cells expressing human TMRPSS2 as previously published^58^. [R.J.W.] RNA was extracted from clarified nasal washes using the Qiagen RNeasy extraction kit (Qiagen, Hilden Germany) following the manufacturer’s instructions. Samples were purified on the included columns and eluted in 50 μL of nuclease free water. PCR was conducted using 4X TaqMan Fast Virus Master Mix (Thermo Fisher) and the HKU *N*-gene primer/probe set.

### Plaque assay.

Vero-TMPRSS2 or Vero-TMPRSS2-hACE2 cells were seeded at a density of 1×10^5^ cells per well in 24-well tissue culture plates. The following day, medium was removed and replaced with 200 μL of material to be titrated diluted serially in DMEM supplemented with 2% FBS. One hour later, 1 mL of methylcellulose overlay was added. Plates were incubated for 72 h, then fixed with 4% paraformaldehyde (final concentration) in PBS for 20 min. Plates were stained with 0.05% (w/v) crystal violet in 20% methanol and washed twice with distilled, deionized water.

### Micro-CT imaging.

Hamsters were inoculated intranasally with 10^3^ PFU (in 30 μL) of B.1.1.529 (strain NC928), B.1.617.2 (UW-5250) or PBS. Lungs of the infected animals were imaged by using an in vivo micro-CT scanner (CosmoScan FX; Rigaku Corporation, Japan). Under ketamine-xylazine anesthesia, the animals were placed in the image chamber and scanned for 2 min at 90 kV, 88 μA, FOV 45 mm, and pixel size 90.0 μm. After scanning, the lung images were reconstructed by using the CosmoScan Database software of the micro-CT (Rigaku Corporation, Japan) and analyzed by using the manufacturer-supplied software. A CT severity score, adapted from a human scoring system, was used to grade the severity of the lung abnormalities^59^. Each lung lobe was analyzed for degree of involvement and scored from 0–4 depending on the severity: 0 (none, 0%), 1 (minimal, 1%–25%), 2 (mild, 26%–50%), 3 (moderate, 51%–75%), or 4 (severe, 76%–100%). Scores for the five lung lobes were summed to obtain a total severity score of 0–20, reflecting the severity of abnormalities across the three infected groups. Images were anonymized and randomized; the scorer was blinded to the group allocation.

### Pathology.

Excised animal tissues were fixed in 4% paraformaldehyde in PBS, and processed for paraffin embedding. The paraffin blocks were cut into 3-μm-thick sections and mounted on silane-coated glass slides. Sections were processed for *in situ* hybridization using an RNA scope 2.5 HD Red Detection kit (Advanced Cell Diagnostics, Newark, California) with antisense probe targeting the nucleocapsid gene of SARS-CoV-2 (Advanced Cell Diagnostics). Specific antigen-antibody reactions were visualized by means of 3,3’-diaminobenzidine tetrahydrochloride staining using the Dako Envision system (Dako Cytomation). Lung tissue sections also were scored based on pathological changes. Scores were determined based on the percentage of alveolar inflammation in a given area of a pulmonary section collected from each animal in each group by using the following scoring system: 0, no pathological change; 1, affected area (≤10%); 2, affected area (<50%, >10%); 3, affected area (≥50%); an additional point was added when pulmonary edema and/or alveolar hemorrhage was observed.

### Data availability.

All data supporting the findings of this study are available within the paper, in the Source Data, and from the corresponding author upon request. There are no restrictions in obtaining access to primary data.

### Code availability.

No code was used in the course of the data acquisition or analysis.

### Reagent availability.

All reagents described in this paper are available through Material Transfer Agreements.

### Statistical analysis.

The number of independent experiments and technical replicates used are indicated in the relevant Figure legends. Statistical analysis included unpaired t tests, Mann-Whitney U tests, and ANOVA with multiple corrections post-tests.
